# The Circular RNA circRNA124534 Promotes Osteogenic Differentiation of Human Dental Pulp Stem Cells Through Modulation of the miR-496/β-Catenin Pathway

**DOI:** 10.3389/fcell.2020.00230

**Published:** 2020-04-03

**Authors:** Fang Ji, Jing Pan, Zhecheng Shen, Zhao Yang, Jian Wang, Xuebing Bai, Jiang Tao

**Affiliations:** ^1^Department of Orthodontics, Ninth People’s Hospital, Shanghai Jiao Tong University School of Medicine, Shanghai, China; ^2^National Clinical Research Center for Oral Diseases, Shanghai Key Laboratory of Stomatology and Shanghai Research Institute of Stomatology, Shanghai, China; ^3^Department of General Dentistry, Ninth People’s Hospital, Shanghai Jiao Tong University School of Medicine, Shanghai, China

**Keywords:** DPSCs, osteogenic differentiation, circular RNA, microRNA, β-catenin

## Abstract

Circular RNAs (circRNAs) have been found to be a crucial role in stem cell-associated bone regeneration. However, the functions and underlying mechanisms of circRNAs in the osteogenic differentiation of human dental pulp stem cells (hDPSCs) remain largely unclear. We found that overexpression of circRNA124534 unexpectedly promoted DPSCs osteogenesis *in vitro* and *in vivo*. Our results confirmed circRNA124534, acting as a miRNA sponge, directly interacts with miR-496 and consequently regulates β-catenin, which in turn exerts osteogenesis of DPSCs. Enforced expression of miR-496 reversed the osteogenesis of circRNA124534, and suppression of miR-496 enhanced the osteogenic differentiation of DPSCs by promoting β-catenin. In conclusion, our findings demonstrate functions of circRNA124534 in modulating osteogenic differentiation through the miR-496/β-catenin pathway; thus, providing a novel potential target for therapy.

## Introduction

Stem cells are a major source in regenerative medicine and are considered to be undifferentiated cells with self-renewal and multi-directional differentiation capability to produce one or more specific cell types. Stem cells can be split in three major categories: embryonic stem cells, induced pluripotent stem cells, and stem cells. According to differentiation potential, stem cells are classified as follows; totipotent, pluripotent, multipotent, oligopotent, and unipotent (Malaver-Ortega et al., 2012). hDPSCs are a multipotent stem cell population resident in dental pulp which are ectodermally isolated cells that originate from migrated neural crest cells which have the same tissue source as craniomaxillofacial and periodontal tissues. They have mesenchymal stem cell characteristics, like fibroblast-like morphology; adherence to culture dish surfaces; formation of colonies in cell culture (Martens et al., 2013); and high proliferation, self-renewing ability, and multi-lineage differentiation probable. Beneath appropriate induction conditions, these cells can differentiate into chondrocytes, fat cells, odontoblasts, and neuron-like cells (Rodriguez-Lozano et al., 2011). In Gronthos et al. (2000) first found that after the separation of DPSCs from the third molar, new types of stem cells can be achieved from various types of perioral tissues, such as human carious deciduous teeth (Zainuri et al., 2018), periodontal ligaments (Seo et al., 2005), the dental follicle of wisdom teeth (Morsczeck et al., 2005), apical papilla (Cantore et al., 2017), and gingiva tissue (Zhang et al., 2009). Moreover, Alge et al. (2010) found DPSCs have higher proliferation rates, cloning potential, and cell numbers than Bone marrow mesenchymal stem cells (BMMSCs). Nevertheless, to ensure the safety and efficacy of DPSCs in clinical applications, we examined the potential for clinical use of DPSCs isolated from human teeth.

circRNAs are a kind of non-linear, non-coding RNAs (ncRNAs) that, unlike conventional linear RNAs, lack a distinctive terminal structure (5′ cap structure and 3′ polyadenylation tail) (Vergani, 1971). In 1976, circRNAs were found in plants and were considered to be a product with incorrect splicing and were not considered important (Jeck and Sharpless, 2014). In the next 30 years, only several reports described circRNAs in mammals. In Salzman et al. (2012), through high-throughput sequencing, determined that thousands genes express circRNAs, and the number and breadth of circRNAs were found to be underestimated. The circRNA CDRlas has multiple miRNA-7 binding sites; acting like spongy to miRNA-7, it competes with miRNA-7 target genes (EGFR, IRS-1, IRS-2, Pak1, Raf1, Ack1, and PIK3CD) for binding to miRNA-7, thus, negatively regulating the activity of miRNA-7 (Hansen et al., 2013). The features and functions of circRNAs are increasingly being understood. CircRNAs, beyond being a class of expression-rich, stable, diverse, and conserved ncRNAs, can act as sponges for miRNAs and participate in controlling gene expression at the translational level, in tumors and cardiovascular and cerebrovascular diseases. CircRNAs are used as clinical biomarkers in various diseases, such as neuropsychiatric diseases (Ebbesen et al., 2016). Although most present studies on circRNAs pay attention to cancer, some articles have indicated that circRNAs are participated in tissue regeneration and stem cell differentiation processes, such as the initial stage of rat liver regeneration (Li et al., 2017a) and osteogenic differentiation of maxillary sinus membrane stem cells (Peng et al., 2019). CircRNAs are highly enriched during human induced pluripotent stem cell differentiation, and cardiac-specific circRNAs can be used as biomarkers of cardiomyocytes (Lei et al., 2018). BMP2 induces osteoblast differentiation through circl9142/circ5846 targeting of miRNAs and mRNAs (Qian et al., 2017). CircRNAs are predicted to function during osteogenic differentiation of periodontal ligament stem cells (Zheng et al., 2017). A human circRNA microarray study has shown that After 7 days of culture in bone marrow stem cells in osteogenic medium, hsa_circ_0124534 (circRNA124534) is significantly upregulated (Zhang et al., 2019). We have previously found that the level of circRNA124534 significantly increases after induction for 2 weeks in osteogenic medium in DPSCs. Hence, our target is to further investigate the mechanism of circRNA124534 in hDPSCs osteogenesis and to suggest potential therapeutic strategies for bone regeneration.

## Results

### Culture and Characterization of DPSCs and Expression of CircRNA124534 in DPSCs

**FIGURE 1:**
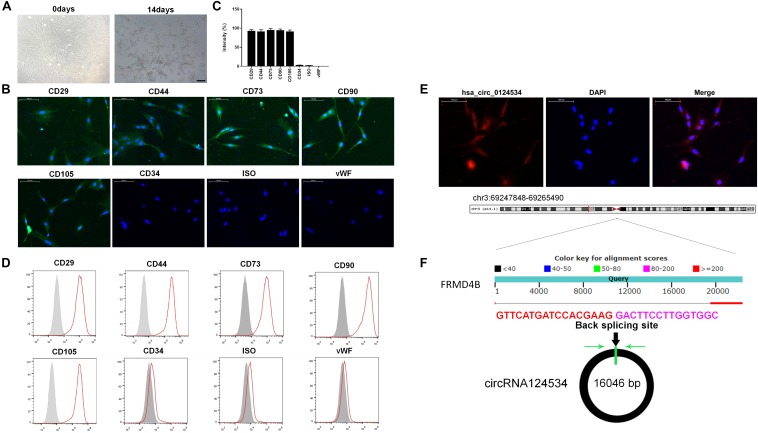
Characteristics of DPSCs and circRNA124534. **(A)** Typical fibroblastic-like morphology of DPSCs at 0 days and 14 days. Scale bars = 100 mm. **(B,C)** Assessment of cell surface markers by immunofluorescence staining and quantified. DPSCs were positive for the markers CD29, CD44, CD73, CD90, and CD105, but negative for the endothelial markers CD34 and von Willebrand factor. Negative isotype controls are shown. Scale bars = 100 mm. **(D)** Flow cytometry analysis of the surface markers in DPSCs. **(E)** FISH was conducted to determine the subcellular localization of circRNA124534 in DPSCs. Scale bars = 100 mm. **(F)** The genomic loci of the FRMD4B gene and circRNA124534. Green arrow indicates back-splicing.

The DPSCs had a fibroblastic-like morphology (Figure 1A). Cell surface markers were used to verify DPSCs cell phenotypes. DPSCs were positive for MSC markers CD29, CD44, CD73, CD90, and CD105, but were negative for CD34 and von Willebrand factor (Figures 1B–D). FISH analysis revealed that circRNA124534 was predominately localized to the cytoplasm (Figure 1E). CircRNA124534 is located on chr3: 69247848–69265490 (Figure 1F), is derived from FRMD4B gene exons, and is 17643 bp in length after splicing.

### CircRNA124534 Promotes the Osteogenic Differentiation of DPSCs and Functions as a miR-496 Sponge in DPSCs

**FIGURE 2:**
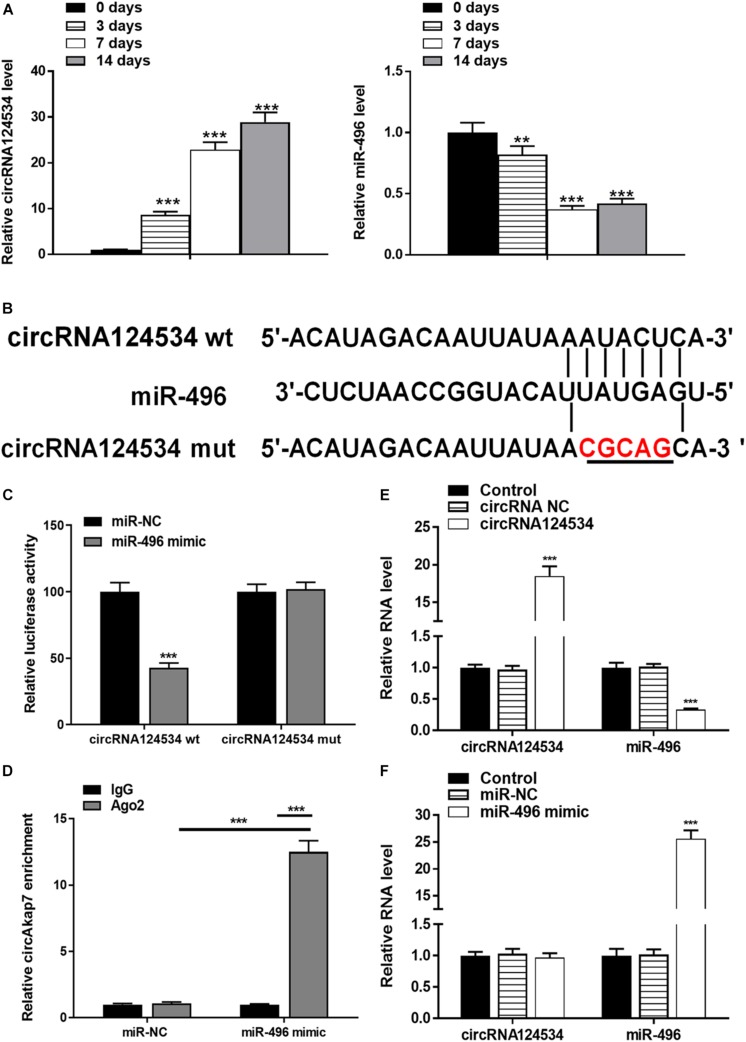
circRNA124534 functions as a miRNA sponge and negatively regulates miR-496 in DPSCs. **(A)** The levels of circRNA124534 and miR-496 were determined by RT-PCR at various time points during the osteogenic differentiation of DPSCs. **(B)** Wild-type (wt) and mutant (mut) circRNA124534 were transfected into DPSCs with or without synthetic miR-496 mimics. **(C)** Relative luciferase activity in DPSCs was detected with luciferase assays. **(D)** Anti-AGO2 RNA immunoprecipitation was performed in DPSCs transfected with miR-496 mimics or miR-NC, and this was followed by RT-PCR to detect circRNA124534. **(E,F)** The levels of circRNA124534 and miR-496 were determined by RT-PCR in DPSCs transfected with circRNA124534 or miR-496 mimic for 48 h. Data indicate the mean ± SD, *n* = 3. **P < 0.01, ***P < 0.001 vs. control.

**FIGURE 3:**
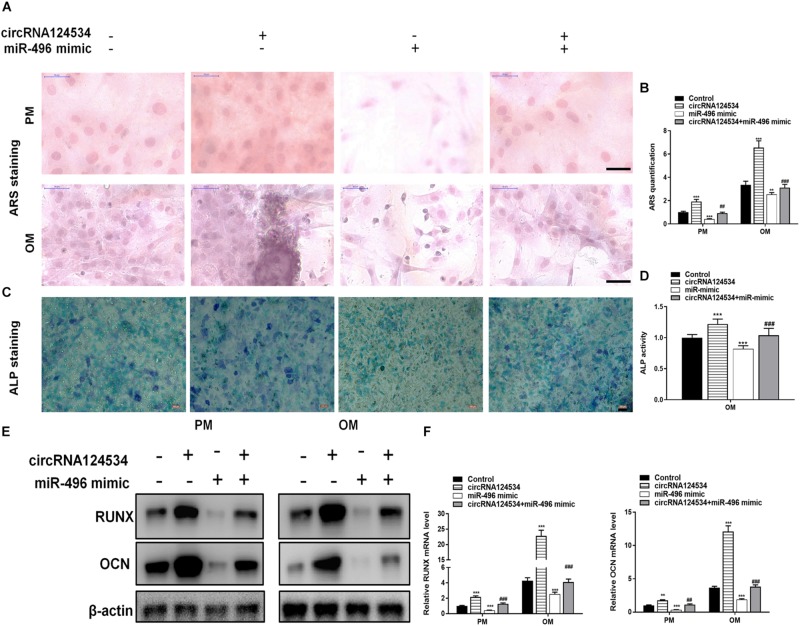
circRNA124534 promotes the osteogenic differentiation of DPSCs *in vitro.* **(A,B)** ARS mineralization assay on day 14 after osteogenic induction in proliferation medium (PM) or osteogenic medium (OM) Scale bars = 50 μm. **(C,D)** ALP activity on day 7 after osteogenic induction. Scale bars = 100 μm. **(E,F)** The levels of RUNX and OCN were determined by RT-PCR and western blotting in DPSCs on day 14 after osteogenic induction. Data indicate the mean ± SD, *n* = 3. **P < 0.01, ***P < 0.001 vs. control, ^##^*P* < 0.01, ^###^*P* < 0.001 vs. circRNA124534.

We evaluated the levels of circRNA124534 and miR-496 during the osteogenic differentiation of DPSCs in virous hours. The expression of circRNA124534 was markedly increased after osteogenic medium induction and maintained high during osteogenic differentiation. Conversely, the level of miR-496 was downregulated during osteogenesis (Figure 2A). Bioinformatics analysis revealed that miR-496 has a binding site for circRNA124534 (Figure 2B). Luciferase reporter assay was performed in DPSCs. enforced expression of miR-496 in DPSCs blocked the activity of the 3′-UTR in the wild-type circRNA124534. However, the activity was not changed in the mutant-type circRNA124534. The outcomes demonstrated that miR-496 is a direct target of circRNA124534 (Figure 2C). In anti-AGO2 RNA immunoprecipitation assays in DPSCs, we used miR-496 mimic to pull down circRNA124534 with anti-AGO2 or control; RT-PCR was performed to evaluate the level of circRNA124534. The circRNA124534 pulled down with anti-AGO2 was enriched in miR-496 mimic group (Figure 2D). These data showed that circRNA124534 functioned as sponge to miR-496. To explore the role of circRNA124534 in DPSCs, DPSCs were transfected with a circRNA124534 overexpression vector or miR-496 mimic for 48 h, then assessed the levels of circRNA124534 and miR-496. RT-PCR indicated that the circRNA124534 overexpression vector upregulated circRNA124534 expression, whereas the level of miR-496 was blocked in DPSCs (Figure 2E). The level of miR-496 in DPSCs significantly increased, whereas circRNA124534 expression did not show clear changes compared with the levels in the control groups after transfection with miR-496 mimic (Figure 2F). ARS staining indicated that circRNA124534 improved the osteogenic differentiation of DPSCs cultured in proliferation medium (PM) or osteogenic medium (OM) on day 14 (Figures 3A,B), whereas miR-469 mimic suppressed the ARS activity. The extracellular mineralization detected by ALP on day 7 showed results same effect of ARS results (Figures 3C,D). Moreover, it is well known that RUNX2 and OCN play essential roles in bone formation. Hence, we evaluated the level of RUNX2 and OCN to verify the effect of circRNA124534 on RUNX2 and OCN. RT-PCR and western blotting results showed that circRNA124534 markedly enhanced the levels of osteogenic markers RUNX2 and OCN, whereas RUNX2 and OCN were downregulated in DPSCs transfected with miR-496 mimic compared with circRNA124534 (Figures 3E,F).

### The Activity of miR-496 in DPSCs Is Mediated by β-Catenin

**FIGURE 4:**
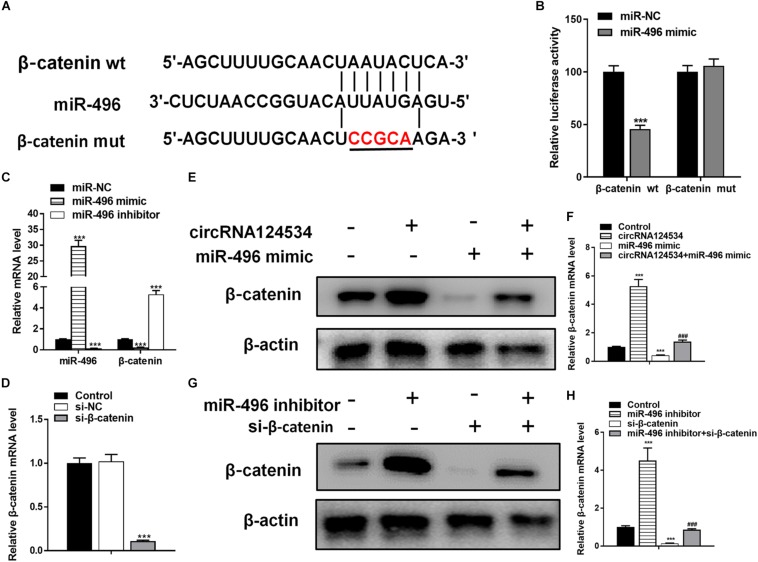
The role of miR-496 in DPSCs is mediated by β-catenin modulation. **(A)** The predicted binding sites of miR-496 in the 3′ UTR of β-catenin. The mutated version of the β-catenin 3′ UTR is also shown. **(B)** The relative luciferase activity was determined in DPSCs 48 h after transfection with the miR-496 mimic/NC or the 3′ UTR of β-catenin wt/mut constructs. **(E,F)** The level of β-catenin was determined by RT-PCR and western blotting in DPSCs transfected with miR-496 mimics or circRNA124534 for 48 h. **(G,H)** The level of β-catenin was determined by RT-PCR and western blotting in DPSCs transfected with miR-496 inhibitor or si-β-catenin for 48 h. Data indicate the mean ± SD, *n* = 3. **(C)** The level of miR-496 and β-catenin was determined by RT-PCR in DPSCs transfected with miR-496 mimics or miR-496 inhibitor for 48 h. **(D)** The level of β-catenin was determined by RT-PCR in DPSCs transfected with si-β-catenin or si-NC inhibitor for 48 h. **P < 0.01, ***P < 0.001 vs. miR-NC **(B–D)** or control **(F,H)**, ^##^*P* < 0.01, ^###^*P* < 0.001 vs. circRNA124534 **(F,H)**.

Bioinformatics analysis have identified β-catenin having a potential binding site to miR-496. β-catenin has been found to be involved in osteogenic differentiation in various stem cells (Gu et al., 2017; Jiang et al., 2017; Xie et al., 2018). Hence, we next assessed the function of β-catenin in DPSCs. To verify whether β-catenin is the target of miR-496, we cloned the wild-type and mutant β-catenin sequences and constructed reporter plasmids and mutant vectors, respectively (Figure 4A). Whereas enforced expression of miR-496 and reporter plasmids visibly decreased the luciferase activity, enforced expression of miR-496 and β-catenin mutated vectors significantly affected the luciferase activity. Hence, the data indicated miR-496 directly targets β-catenin (Figure 4B). Next, we transfected miR-496 inhibitor into DPSCs. The RT-PCR results indicated that miR-496 inhibitor downregulated the expression of miR-496 and upregulated the expression of β-catenin, whereas miR-496 mimic upregulated the expression of miR-496 and downregulated the expression of β-catenin significantly (Figure 4C). To further investigate the role of β-catenin in DPSCs, we constructed a small interfering RNA targeting β-catenin (si-β-catenin). As expected, the RT-PCR data showed that si-β-catenin significantly downregulated β-catenin (Figure 4D). Furthermore, we used RT-PCR and western blotting to assess the mRNA and protein levels of β-catenin. Overexpression of circRNA124534 markedly enhanced the levels of β-catenin. β-catenin was markedly repressed in DPSCs transfected with miR-496 mimic or si-β-catenin, while it was increased in miR-496 inhibitor group (Figures 4E–H).

**FIGURE 5:**
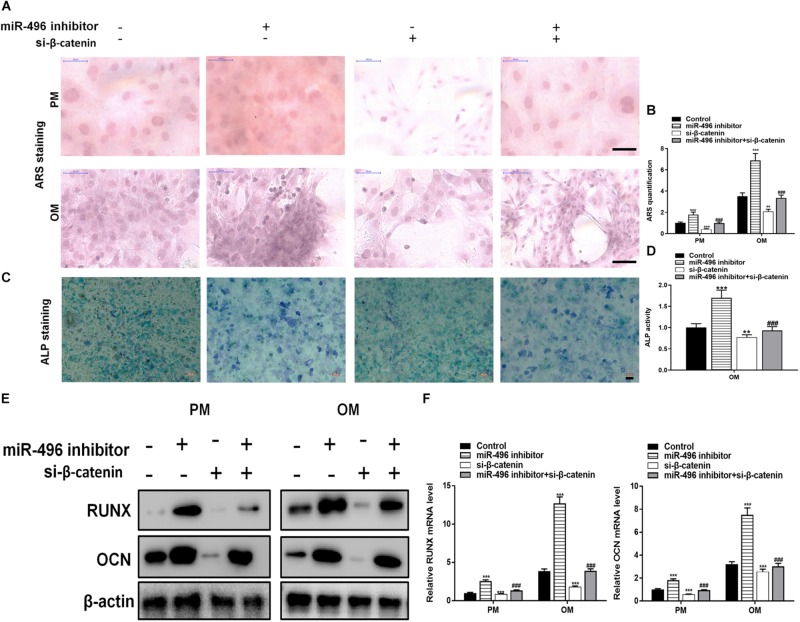
Inhibition of miR-496 promotes the osteogenic differentiation of DPSCs *in vitro.* **(A,B)** ARS mineralization assay on day 14 after osteogenic induction in PM or OM. Scale bars = 50 μm. **(C,D)** ALP activity on day 7 after osteogenic induction. Scale bars = 100 μm. **(E,F)** The levels of RUNX and OCN were determined by RT-PCR and western blotting in DPSCs on day 14 after osteogenic induction. Data indicate the mean ± SD, *n* = 3. **P < 0.01, ***P < 0.001 vs. control, ^###^*P* < 0.001 vs. miR-496 inhibitor.

Next, ARS staining indicated that the miR-496 inhibitor stimulated osteogenic differentiation of DPSCs cultured (Figures 5A,B), whereas si-β-catenin suppressed the ARS activity. ALP on day 7 showed results same effect of ARS results (Figures 5C,D). Moreover, miR-496 inhibitor markedly enhanced the levels of osteogenic markers RUNX2 and OCN, which were detected by RT-PCR and western blotting, whereas RUNX2 and OCN were lower in DPSCs transfected with si-β-catenin than with the miR-496 inhibitor (Figures 5E,F).

### Silencing CircRNA124534 Suppresses the Osteogenic Differentiation of DPSCs and Overexpressing β-Catenin Reversed the Inhibition

**FIGURE 6:**
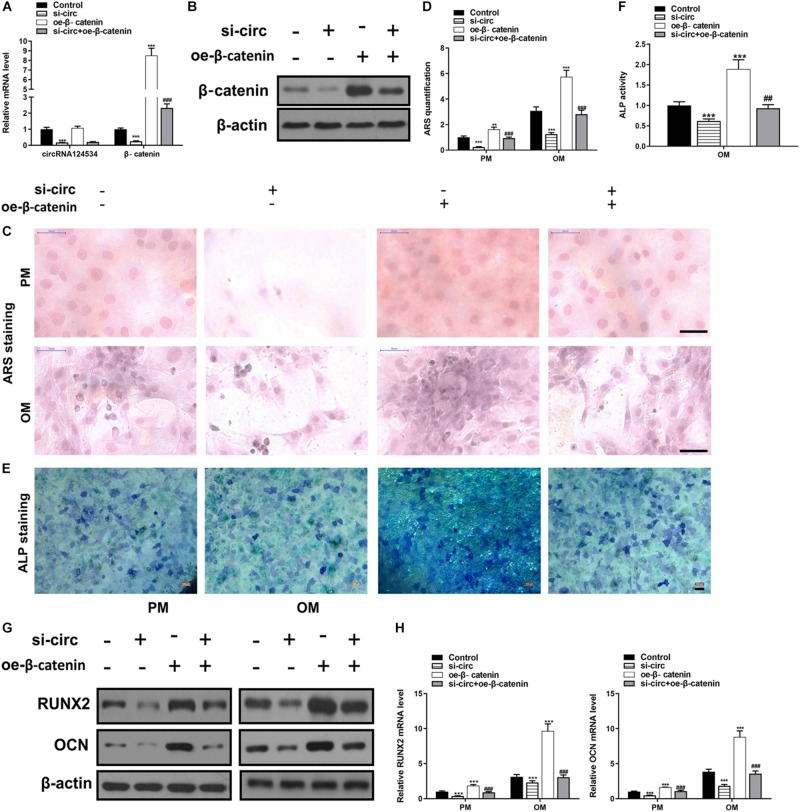
Silencing CircRNA124534 suppresses the osteogenic differentiation of DPSCs and overexpressing β-catenin reversed the inhibition. **(A)** The level of CircRNA124534 and β-catenin was determined by RT-PCR in DPSCs transfected with CircRNA124534 silencing vector (si-circ) or β-catenin overexpression vector (oe-β-catenin) for 48 h. **(B)** The level ofβ-catenin was determined by western blotting in DPSCs transfected with si-circ or oe-β-catenin for 48 h. **(C,D)** ARS mineralization assay on day 14 after osteogenic induction in PM or OM. Scale bars = 50 μm. **(E,F)** ALP activity on day 7 after osteogenic induction. Scale bars = 100 μm. **(G,H)** The levels of RUNX and OCN were determined by RT-PCR and western blotting in DPSCs on day 14 after osteogenic induction. Data indicate the mean ± SD, *n* = 3. **P < 0.01, ***P < 0.001 vs. control, ^##^*P* < 0.01, ^###^*P* < 0.001 vs. miR-496 inhibitor.

CircRNA124534 silencing vector (si-circ) or β-catenin overexpression vector (oe-β-catenin) were transfected into DPSCs. RT-PCR and western blotting data showed that Silencing CircRNA124534 significantly inhibited the level of β-catenin while overexpressing β-catenin did not alter the level of CircRNA124534 (Figures 6A,B) in DPSCs. Furthermore, ARS staining and ALP staining indicated CircRNA124534 downregulation markedly suppresses the osteogenic differentiation of DPSCs. However, overexpression of β-catenin reversed this suppression and promoting the osteogenic differentiation of DPSCs (Figures 6C–F). Moreover, Silencing CircRNA124534 markedly restrained the levels of osteogenic markers RUNX2 and OCN, which were detected by RT-PCR and western blotting, whereas RUNX2 and OCN were higher in DPSCs in oe-β-catenin group (Figures 6G,H).

### CircRNA124534 Enhances Osteogenic Differentiation of DPSCs *in vivo*

**FIGURE 7:**
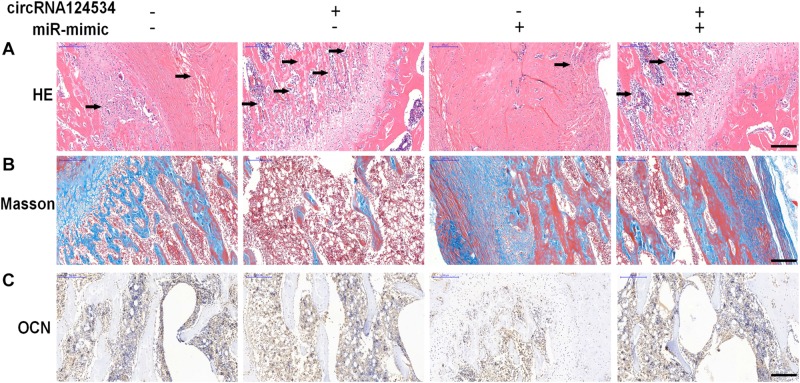
circRNA124534 enhanced the osteogenic differentiation of DPSCs *in vitro*. **(A)** Representative image of H&E staining and trabecular boneare marked with arrows. Scale bars = 200 mm. **(B)** Representative image of Masson’s trichrome staining. Scale bars = 200 mm. **(C)** Representative image of IHC staining for OCN. Scale bars = 200 mm.

To investigate whether circRNA124534 promotes bone formation in mice model, we loaded DPSCs transfected with circRNA124534 overexpression or miR-496 mimic vectors onto scaffolds and implanted them in the subcutaneous space in nude mice (*n* = 6). After 8 weeks, the implantation samples were harvested, embedded in paraffin sections, deparaffinized, and stained with H&E and Masson’s trichrome stain, and subjected to immunohistochemistry (IHC). H&E staining showed little bone formation in the miR-496 mimic group, whereas osteoid formation was observed in the circRNA124534 group (Figure 7A). Collagen organization with blue color in Masson’s trichrome staining was significantly increased in the circRNA124534 group and decreased in the miR-496 mimic group (Figure 7B). Because OCN is a marker of osteogenesis, we assessed the intensity of OCN with IHC assays. The intensity of staining was enhanced in the circRNA124534 group and suppressed in the miR-496 mimic group (Figure 7C).

## Discussion

In recent years, with the progression of circRNA research, many researches have reported the participation of circRNAs in the progression of various diseases, including arteriosclerosis (Burd et al., 2010), nervous system disorders (Li et al., 2017b; Akhter, 2018), aging (Westholm et al., 2014), heart failure (Wang et al., 2016), and cancers (Qu et al., 2017; Zhang et al., 2018). Yet, its effect and mechanism in osteogenic differentiation requires more exploration. We found that circRNA124534 plays a pivotal role in osteogenesis. The expression of circRNA124534 increased more than 30-fold after induction for 14 days in osteogenic medium in DPSCs. Overexpression of circRNA124534 enhanced DPSC osteogenic differentiation both *in vitro* and *in vivo*. circRNA124534 can act as a sponge by absorbing miR-496 and consequently reverses the repressive function on the β-catenin target. Thus, our data showed that circRNA124534 has a vital function in the pathogenesis and development of osteogenesis. However, there are some limitations in our present research. Several studies have reported that in bone formation, injured bones tend to show unusual inflammatory responses (Bastian et al., 2011). Bone regeneration is compromised under pathogenic conditions of chronic inflammation (Krum et al., 2010). Nevertheless, the connection between inflammation and the level of circRNA124534 in DPSC osteogenic differentiation must be confirmed in future studies.

Several studies have shown that abnormal expression of miRNAs can affect bone metabolism, which in turn affects bone development and bone shaping processes. Eskildsen et al. (2011) has found that when bone marrow mesenchymal stem cells differentiate into osteoblasts, the level of miR-138 is decreased, and overexpression of miR-138 inhibits osteogenic differentiation. Inhibition of miR-138 expression has revealed increased expression of OCN and increased activation of ALP. Chen et al. (2017) examined the induction of human adipose-derived mesenchymal stem cells. During osteogenic differentiation, miR-375 levels rise; overexpression of miR-375 promotes cell osteogenesis, and specific knockout of miR-375 inhibits cell differentiation into bone cells. Therefore, miRNAs play an important role in the regulation of stem cell osteogenic differentiation. Aberrant expression of miR-125a-5p has been observed in various diseases, including myocardial infarction (Jin and Ni, 2019), atherosclerosis (Hu et al., 2019), cerebral injury (Yao et al., 2019), diabetes (Rubie et al., 2019), and various malignant tumors (Mason et al., 2018; Ma et al., 2019; Wang et al., 2019). Inhibition of miR-496 has been found to reverse the inhibitory effects of IL-1β in bone marrow stem cell-associated bone regeneration (Huang and Chen, 2017). In our study of miRNA, miR-496 was markedly downregulated with overexpression circRNA124534 in DPSCs compared with control DPSCs. According to the starBase v3.0 (Li et al., 2014) predictions, and as demonstrated through luciferase reporter assays, we also found that circRNA124534 and the β-catenin 3′ UTR share identical miR-496 responses and competitively bind miR-496. Furthermore, circRNA124534 binds directly to miR-496 in an AGO2-dependent manner. We confirmed that circRNA124534 can serve as a competitive endogenous RNA element and “sponge” miR-496 during DPSCs osteogenic differentiation.

The Wnt/β-catenin pathway is involved in bone formation and bone regeneration (Lerner and Ohlsson, 2015). Blocking the Wnt/β-catenin pathway often leads to osteogenic differentiation inhibition and bone homeostasis (Baron and Kneissel, 2013). After activation of the Wnt/β-catenin signaling pathway, transcriptional activation of downstream CyclinDl, Runx2, and other target genes can regulate physiological activities, such as cell proliferation and bone differentiation (Kleber and Sommer, 2004; Wilusz and Majka, 2008). β-catenin has been shown to regulate osteogenic differentiation of DPSCs (Lu et al., 2019). In the current study, we showed that miR-496 interacts with the 3′ UTR of β-catenin and suppresses β-catenin activity at the post-transcriptional level. Knockdown of miR-496 suppressed the level of β-catenin and repressed DPSCs osteogenic differentiation.

In conclusion, our study indicates that circRNA124534 plays a novel role in osteogenesis by sponging miR-496. Finally, we discovered that a circRNA124534/miR-496/β-catenin axis may serve as a potential therapeutic target for enhancing bone formation; thus, suggesting the feasibility of circRNA-targeted therapeutic methods in bone tissue engineering.

## Materials and Methods

### Cell Culture and Induction

hDPSCs were purchased from the Shanghai Institutes for Biological Sciences. hDPSCs were obtained from healthy pulp tissues isolated from caries-free teeth of patients (3 females, age 22∼33 years; 3 males, age 26∼41 years) undergoing extraction of fully erupted third molars. The cells were cultured in α-modified Eagle’s medium supplemented with 10% fetal bovine serum (Gibco, Grand Island, NY, United States) and 1% penicillin/streptomycin at 37°C in a humidified atmosphere of 5% CO_2_ and 95% air. For Immunofluorescence: The hDPSCs were fixed with 4% paraformaldehyde (PFA) for 10 min, followed by blocking with 5% BSA in PBS for 60 min at room temperature. The cells were incubated with the following antibodies at room temperature for 1 h: rabbit anti-CD29, CD44, CD73, CD90, and CD105 (1:200; Abcam Inc.). Following a wash in PBS, the cells were incubated in goat anti-rabbit secondary antibodies conjugated with FITC (1:200; Abcam) in PBS for 1 h at room temperature. Isotype-identical antibodies (PharMingen, San Diego, CA, United States) served as the controls. DAPI was used for the nuclear stain. Images were obtained using an inverted fluorescence microscope. For Flow cytometer: hDPSCs were identified and selected by flow cytometry (FCM) with anti-CD29, CD44, CD73, CD90, and CD105 (1:200; Abcam Inc.). After being subcultured to the third generation, hDPSCs at 80% confluence were washed twice with PBS followed by digestion with 0.25% trypsin–EDTA (Thermo Fisher Scientific, United States). The cells were then centrifuged at 1,000 rpm and washed with PBS. After incubation with antibodies and their isotype controls (1:100) (PharMingen, San Diego, CA, United States) at 4°C for 30 min, the cells were flowed through the cytometer at about 1,000 cells per second. Results of FCM were analyzed by FlowJo software (FlowJo, LLC, United States). The osteogenesis of hDPSCs was assayed after 4 passages and in cells seeded in 96-well plate at density of 10,000 cells/cm^2^. Osteogenic differentiation was induced by changing the medium to osteogenic medium containing 10%FBS, 0.1 μM dexamethasone, 50 μg/mL L-ascorbic acid and 10 mM β-glycerophosphate (Sigma-Aldrich, St. Louis, MO, United States) when the cells were ∼80% confluent. The medium was replaced every 2 days during the incubation period.

### Fluorescence *in situ* Hybridization (FISH)

Specific probes tailored to the circRNA124534 sequence and labeled with cy5 were used for *in situ* hybridization, as previously described (Zeng et al., 2018). Nuclei were counterstained with 4,6-diamidino-2-phenylindole. All procedures were conducted according to the manufacturer’s protocol (GenePharma, Shanghai, China).

### CircRNA Analysis and Target Prediction

Prediction of the hsa_circ_0124534-miRNA-target genes was performed with the STARBASE website^1^ (Li et al., 2014).

### Luciferase Reporter Assay

DPSCs were co-transfected with plasmids containing the 3′-UTR of wild-type or mutant fragments from β-catenin, or the predicted binding sequence of circRNA124534 and miR-496 mimics, by using Lipofectamine 2000 (Invitrogen, MA, United States), according to the manufacturer’s protocol. Forty-eight hours after transfection, firefly and Renilla luciferase activity was measured consecutively with a dual-luciferase reporter assay system (Promega, Fitchburg, WI, United States). Luminescence ratios of firefly to Renilla luciferase were calculated, and each assay was independently repeated in triplicate.

### Cell Transfection

To assess circRNA124534 expression, we obtained circRNA124534 overexpression vectors constructed by GenePharma (Shanghai, China). DPSCs were then electroporated with 50 nM of circRNA124534 overexpression vector with Nepa21 pulse generator (NepaGene, Chiba, Japan).

To assess miR-496 expression, we obtained a miR-496 overexpression vector (miR-mimic) and negative control (miR-NC) created by GenePharma. Subsequently, DPSCs were transfected with either the miR-496 overexpression construct or miR-NC at a concentration of 50 nM with Lipofectamine 2000 (Invitrogen). Cells were used for miR-496 expression analysis or other experiments 48 h after transfection. For miR-496 inhibition analysis, glioma cells were treated with a miR-496 inhibitor for 48 h before miR-496 expression analysis or other experiments were performed.

For β-catenin inhibition, an si-β-catenin vector was constructed by GenePharma. DPSCs were transfected with either 50 nM of the si-β-catenin vector or the negative control with Lipofectamine 2000 (Invitrogen). All steps were performed according to the manufacturer’s protocol.

### Alizarin Red S (ARS) Staining and Quantification

After osteogenic cultivation for 14 days, DPSCs were fixed in 95% ethanol for 0.5 h at room temperature. Then, DPSCs were washed with distilled water and stained with 0.1% ARS (pH 4.2; Sigma-Aldrich) for 20 min. To evaluate the mineralized nodules, we dissolved the stain in 1 mL 10% cetylpyridinium chloride (Sigma-Aldrich) for 1 h, and the absorbance at 570 nm was assessed by spectrophotometric methods. The intensity of ARS was normalized to the total protein concentration.

### Alkaline Phosphatase (ALP) Staining and Activity

ALP staining was performed with an NBT/BCIP staining kit (CoWin Biotech). DPSCs were fixed in 4% paraformaldehyde for 10 min, and then ALP staining was performed according to the manufacturer’s protocol. The total protein content was determined in the same sample by Pierce Protein Assay Kit (Thermo Fisher Scientific). ALP activity relative to the control treatment was calculated after normalization to the total protein content.

### RT-PCR Analysis

We extracted total RNA through Trizol reagent (Invitrogen, Carlsbad, CA, United States). We utilized RNase-free DNase Set (Qiagen) to erase genomic DNA contamination. We reverse transcribed1 μg of total RNA through reverse transcriptase (Applied Biosystems, Foster City, CA, United States) and random primers for cDNA synthesis. Afterward, we performed RT-qPCR via Power SYBR Green PCR Mastermix (TaKaRa, Tokyo, Japan) on Applied Biosystems 7500 Real-time Fast PCR System. We carried out PCR in triplicate for every gene. We calculated relative expression by the 2_–ΔΔCt_ method, with glyceraldehyde-3-phosphate dehydrogenase (GAPDH) or U6 for normalization. Table 1 lists Human gene-specific PCR primers.

**TABLE 1:** The primers used in this study.

**Gene name**	**Forward (5′-3′)**	**Reverse (5′-3′)**
RUNX2	ACTACCAGCCACCGAGACCA	ACTGCTTGCAGCCTTAAATGA
		CTCT
OCN	AGCCACCGAGACACCATGAGA	GGCTGCACCTTTGCTGGACT
miR-496	CGCTGAGTATTACATGGCCAA	CAGTGCGTGTCGTGGAGT
	TCTC	
hsa_circ_	TGAGCTTGTGAGTGAGTGGT	GCAAGGAGAATGGCGAGATG
0124534		
U6	CTCGCTTCGGCAGCACA	AACGCTTCACGAATTTGCGT
GAPDH	CGACAGTCAGCCGCATCTT	CCAATACGACCAAATCCGTTG

### Protein Isolation and Western Blot Analysis

Cells were lysed using RIPA (Sigma-Aldrich) buffer containing a mixture of protease inhibitors. Protein concentrations in cells lysates were determined using a BCA (Thermo Fisher Scientific) kit. Protein from lysed cells (50 μg) was separated by 10% SDS-PAGE and transferred to nitrocellulose membranes, and this was followed by blocking for 2 h. Next, membranes were incubated overnight with primary antibodies followed by horseradish peroxidase-conjugated secondary antibodies. Antibodies for immunoblotting, including β-actin (ab8226, 42 kDa), Runx2 (ab92336, 57 kDa), OCN (ab2360486, 11 kDa), and β-Catenin (ab16051, 94 kDa) were purchased from Abcam (all 1: 1,000 dilutions). The protein bands were visualized with ECL Plus Detection Reagent (Applygen, Beijing, China).

### RNA Immunoprecipitation

According to the manufacturer’s protocol, RNA immunoprecipitation (RIP) was performed in DPSCs cells 48 h after transfection with the miR-496 overexpression construct or miR-NC using the Magna RIPTM RNA Binding Protein Immunoprecipitation Kit (Millipore). Cells (1 × 10^5^) were lysed in RNA lysis buffer, then the cell lysate was conjugated to magnetic beads conjugated to human anti-Argonaute 2 (AGO2) antibody (Millipore) or control mouse IgG (Millipore) in RIP immunoprecipitation buffer. The samples were incubated with proteinase K (Gibco, Grand Island, NY, United States) and IP RNA was isolated. The extracted RNA was examined by reverse transcription PCR to investigate the enrichment of circRNA124534.

### Animals and Cell Transplantation

DPSCs at the fourth passage were infected with lentivirus (circRNA124534 or miR-496 mimic) and cultured in OM for 7 days before the *in vivo* studies. After being trypsinized and resuspended directly in DMEM, DPSCs were cultured with synthograft (β-tricalcium phosphate; Bicon) for 1 h at 37°C, then centrifuged at 150 × g for 5 min, and implanted into two symmetrical sites in the dorsal subcutaneous space in 6-week-old BALB/c nude mice (*n* = 6). All animal experiments were approved by the Ethical Review Committee of Shanghai Jiao Tong University School of Medicine.

### Evaluation of Bone Formation *in vivo*

Specimens were harvested 8 weeks after transplantation, and the mice were euthanized by CO_2_ asphyxiation. The specimens were then decalcified in 10% EDTA (pH 7.4), dehydrated, and embedded in paraffin. Sections (5 μm thickness) were cut and stained with H&E and Masson’s trichrome. For immunohistochemical staining, the sections were permeabilized with 0.1% Triton X-100 in PBS for 15 min, blocked with 3% BSA (Sigma-Aldrich) in PBS for 30 min, and thereafter incubated with a primary antibody against OCN (Abcam) at 4°C overnight. Next, sections were processed using the ABC detection kit (Vector Laboratories). Tissue slices were visualized under a light microscope (Olympus).

### Statistical Analysis

All data are reported as the mean ± SD. One-way analysis of variance was used for multiple group testing. A *p*-value < 0.05 was considered statistically significant.

## Data Availability Statement

The datasets generated for this study are available on request to the corresponding author.

## Ethics Statement

The animal study was reviewed and approved by the Ethical Review Committee of Shanghai Jiao Tong University School of Medicine.

## Author Contributions

FJ was responsible for collection and assembly of data analysis, interpretation, and manuscript writing. JP and XB contributed to the collection and assembly of data analysis and interpretation. ZS, ZY, and JW were responsible for data collection. JT were responsible for conception, design, manuscript revising and confirmation, and financial support. All authors have read and approved the final version of the manuscript.

## Conflict of Interest

The authors declare that the research was conducted in the absence of any commercial or financial relationships that could be construed as a potential conflict of interest.

## Footnotes

1 http://starbase.sysu.edu.cn/
